# High-Temperature Dielectric Relaxation Behaviors in Mn_3_O_4_ Polycrystals

**DOI:** 10.3390/ma12244026

**Published:** 2019-12-04

**Authors:** Songwei Wang, Xin Zhang, Rong Yao, Liguo Fan, Huaiying Zhou

**Affiliations:** 1 School of Materials Science and Engineering, Guilin University of Electronic Technology, Guilin 541004, China; swwang666@126.com (S.W.); Z19877333362@163.com (R.Y.); 15803462571@163.com (L.F.); zhy@guet.edu.cn (H.Z.); 2 Guangxi Key Laboratory of Information Materials, Guilin University of Electronic Technology, Guilin 541004, China

**Keywords:** dielectric relaxation, activation energy, oxygen vacancies hopping, positive temperature coefficient of resistance (PTCR) effect

## Abstract

High temperature dielectric relaxation behaviors of single phase Mn_3_O_4_ polycrystalline ceramics prepared by spark plasma sintering technology have been studied. Two dielectric relaxations were observed in the temperature range of 200 K–330 K and in the frequency range of 20 Hz–10 MHz. The lower temperature relaxation is a type of thermally activated relaxation process, which mainly results from the hopping of oxygen vacancies based on the activation energy analysis. There is another abnormal dielectric phenomenon that is different from the conventional thermally activated behavior and is related to a positive temperature coefficient of resistance (PTCR) effect in the temperature region. In line with the impedance analyses, we distinguished the contributions of grains and grain boundaries. A comparison of the frequency-dependent spectra of the imaginary impedance with imaginary electric modulus suggests that both the long range conduction and the localized conduction are responsible for the dielectric relaxations in the Mn_3_O_4_ polycrystalline samples.

## 1. Introduction

The relation between physical properties and microstructure (such as grains, grain boundaries, sample-electrode interfaces, and so on) is an important aspect for ceramic materials and is helpful for better understanding their electrical properties [1,2,3,4,5]. Dielectric, modulus, and impedance measurements are the most widely used characterization methods for investigating the microstructure-property relation and relaxation mechanism. According to the temperature and frequency dependence of the dielectric peaks, the nature of the anomalies may be attributed to a thermally activated behavior, a ferroelectric phase transition, or other mechanisms. Furthermore, the contributions of grains, grain boundaries, and sample-electrode interfaces can be distinguished by impedance spectrum analysis [1,2,3,4].

The PTCR (positive temperature coefficient of resistance) effect is characterized by an increase in resistance with temperature, which is in contrast to the thermally activated behavior in which resistance decreases with temperature. The papers concerning the study of PTCR mainly focus on BaTiO_3_ and donor-doped BaTiO_3_ [6,7,8]. Goodman pointed out that the PTCR effect in BaTiO_3_ was related to the grain boundary [6]. Sinclair et al. suggested that the PTCR effect in BaTiO_3_ stems from the resistances of the grain and grain boundary [3]. The PTCR effect of the donor-doped BaTiO_3_ was demonstrated by Heywang-Jonker model [7,8] and was attributed mainly to the donor dopants, which resulted in the difference of the resistances between the grain and grain boundary.

The strong couplings among spin, charge, lattice, and orbital have received much attention in strongly correlated Mn_3_O_4_ systems. In addition, Mn_3_O_4_ is widely used in the electronics industry and is a raw material for the production of soft magnetic oxyferrites [9,10,11,12,13,14]. The dielectric and magneto-dielectric effects in low temperature (<43 K) for Mn_3_O_4_ have been studied in a few reports [15,16,17,18]. Previous studies indicated that the low temperature magnetoelectric coupling mechanism originated from spin-phonon coupling or the modulation of Mn^3+^ orbital states through the inverse process of single-ion spin anisotropy [15,16]. In this paper, we studied the microstructure-property relation of Mn_3_O_4_ polycrystalline sample in the high temperature range (200–330 K) and demonstrate the comprehensive understanding of dielectric relaxation of Mn_3_O_4_ ceramics by using dielectric and impedance spectroscopy. The results show there are two types of relaxations. The relaxation at lower temperature is a normal thermally activated relaxation process, which is associated with the hopping of oxygen vacancies. The relaxation at higher temperature is attributed to the PTCR effect, caused by the difference of the resistances between the grain and grain boundary. This work is helpful for understanding the dielectric relaxation behaviors in manganese oxides materials.

## 2. Experimental

Single-phase Mn_3_O_4_ polycrystalline samples were prepared using spark plasma sintering technology by adjusting the sintering temperature, applied static pressure, and holding time [19]. Chemical composition and the elemental maps were measured by a Quanta 450 FEG field emission scanning electron microscope (FESEM) and the available energy-dispersive X-ray spectroscopy (EDX) equipment (FEI, Hillsboro, OR, USA). The sintered pellet was polished and then coated with silver glue. The permittivities of samples were measured using a precise impendence analyzer (Wayne Kerr Electronics 6500B, Cavendish Square, London) with an applied voltage of 1 V in the temperature range from 200 K to 330 K and in the frequency range of 20 Hz–10 MHz. The temperature was controlled by a physical properties measurement system (Quantum Design 9T, San Diego, CA, USA).

## 3. Results and Discussion

**Figure 1:**
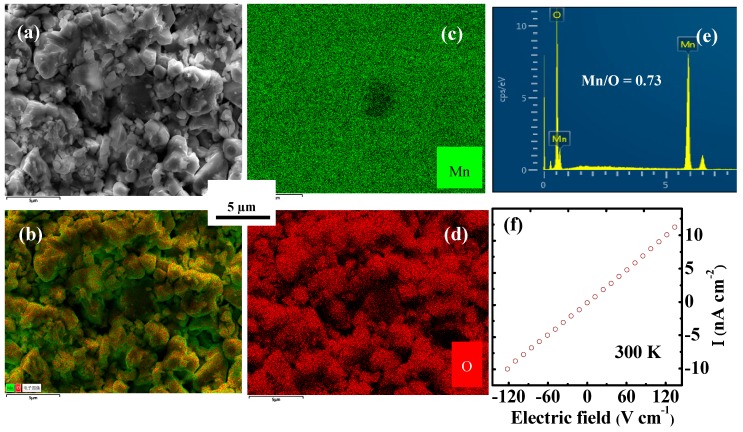
(Color online) (**a**) electronic image, (**b**–**d**) X-ray mapping, (**e**) energy-dispersive X-ray spectroscopy (EDX) spectrogram and (**f**) current-density versus electric-field curve at 300 K of Mn_3_O_4_ polycrystalline sample.

The X-ray diffraction patterns of the Mn_3_O_4_ polycrystalline samples exhibit the single phase character as reported previously [18]. In order to further determine the chemical compositions and elemental maps, the EDX and energy dispersive X-ray analysis (EDXA) spectra measurements were carried out, as shown in Figure 1. The results show only Mn and O elements present in the as-prepared sample and the ratio of Mn:O *=* 0.73 ± 0.006, which is further evidence that the prepared Mn_3_O_4_ has a single phase. We also measured the current-density versus electric-field curve of the sample with silver electrode, as shown in Figure 1f. The nearly linear slope indicates the electrode is a good ohmic contact with ceramics. The silver glue as an electrode has some influence on the dielectric properties, which is helpful for studying these properties.

**Figure 2:**
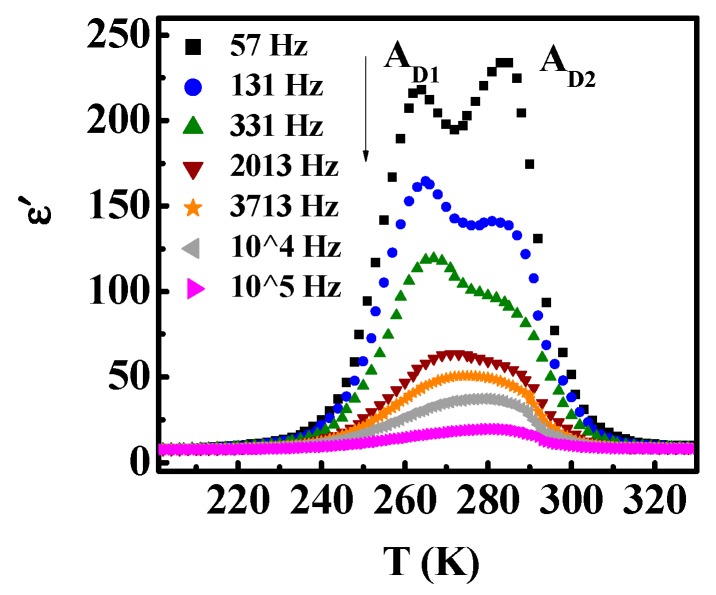
(Color online) Temperature dependence of *ε’* for Mn_3_O_4_ polycrystalline sample measured with various frequencies.

**Figure 3:**
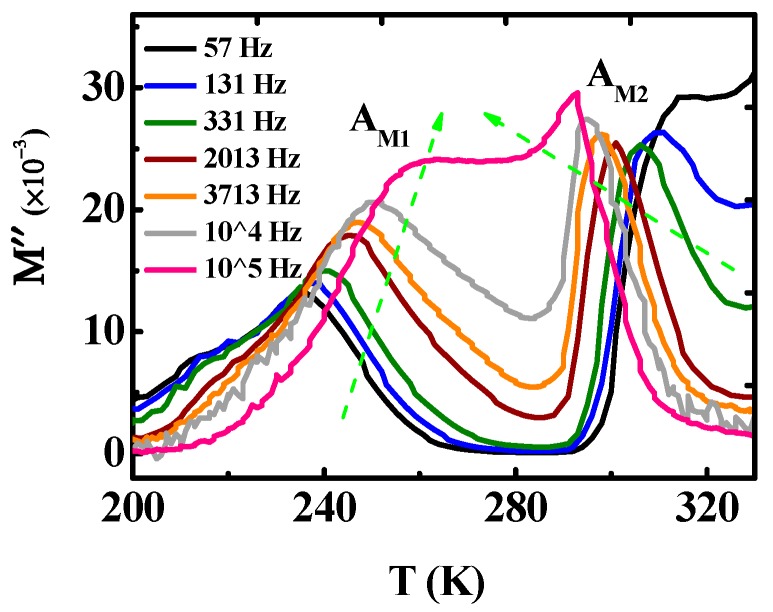
(Color online) The electric modulus imaginary part (*M”*) versus the temperature plots at different frequencies.

Figure 2 shows the temperature dependence of the real part (*ε’*) of the complex dielectric constant (å^*^) at various frequencies for Mn_3_O_4_ polycrystalline sample. There are two dielectric relaxation peaks. The peak at lower temperature moves slightly to the higher temperature with the frequency increasing. The other peak position at higher temperature is almost unaffected by the frequency. The electric modulus can be expressed as M^*^ = 1/å^*^, which suggests that the modulus can largely reduce the background and provide information about the relaxation mechanism [20,21]. Figure 3 shows the temperature dependence of the imaginary part of the modulus (*M”*). The *M”*(*T*) curve shows two pronounced relaxations, from low temperature to high temperature, marked as *A_M1_* and *A_M2_*, respectively. As the frequency increase, *A_M1_* shifts to higher temperature, which indicates a well-known thermally activated behavior. However, the *A_M2_* peak, which is different from the general thermal activation behavior, shifts to lower temperature with the frequency increasing. Therefore, we refer to it as an abnormal thermally activated behavior.

**Figure 4:**
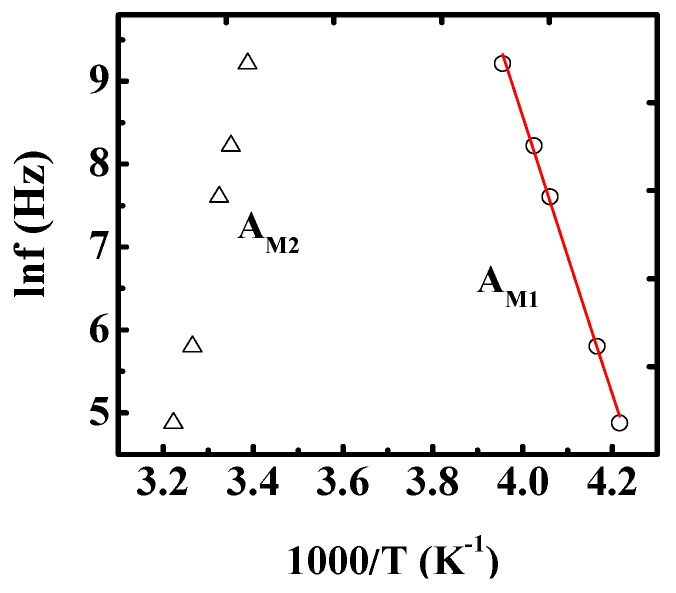
(Color online) Arrhenius plots of *M”* for two types of relaxations (*A_M1_* and *A_M2_*). Symbols are the experimental points and solid line represents the fitting.

Generally speaking, for a thermally activated relaxation process, the variation of peak position can be described by the Arrhenius law [22]:

ƒ = ƒ_0_ exp (−*E_a_*/*k_B_T_M_*) (1)

where ƒ_0_ is pre-exponential and *E_a_* is the activation energy. According to the Arrhenius law, it is clear that lnƒ is proportional to 1/*T_M_*. The activation energy can be obtained according to the slope. We can make a preliminary judgment on the mechanism of relaxation peaks based on the activation energy. Figure 4 shows the Arrhenius plots of *M”* for the two types of relaxations (*A_M1_* and *A_M2_*). The solid line shows the fitting to the experiment data of *A_M1_* by Equation (1). The activation energy was derived to be about 1.44 eV. Similar results were also reported in SrTiO_3_ ceramics [23], PbZr_1−__x_Ti_x_O_3_ single crystals [24], and Mg doped PZT [25], etc. The type of dielectric relaxation is attributed to the mobility of oxygen vacancies [20,26,27]. Therefore, *A_M1_* can be ascribed to the hopping of oxygen vacancies. For the abnormal dielectric relaxation *A_M2_*, the peak position as a function of frequency seems also to follow the Arrhenius law mathematically, but the derived value of *E_a_* is −2.31 eV. Activation energy is the energy required to move a crystal atom away from an equilibrium position to another new equilibrium or unbalanced position. That is to say it is the energy needed to be overcome in order to start a physicochemical process. Therefore, it is difficult to understand a negative value of active energy.

**Figure 5:**
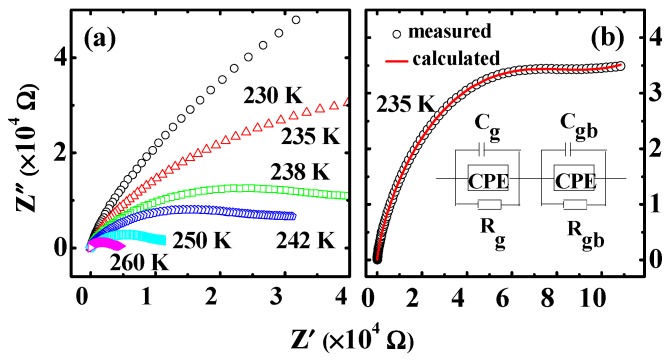
(Color online) (**a**) Complex impedance below 260 K. (**b**) Nyquist plots at 235 K for the circuit shown.

Impedance spectrum analysis is a common method for analyzing the contributions of different microstructural components to the relaxation in ceramic materials [4,28]. In order to get a deep insight into the nature of the relaxation process, the impedance spectrum has been studied. Figure 5a shows the imaginary part of the impedance *Z”* versus the imaginary part *Z’* of the impedance plots (Nyquist plots) below 260 K (for *A_M1_*). The irregular semicircular arc radius decreases with the temperature increasing, which indicates that Mn_3_O_4_ ceramics have smaller resistivity at higher temperatures between 230 K and 260 K. The irregular semicircular coil may suggest the existence of multiple relaxations in a Mn_3_O_4_ polycrystalline sample [4]. The Nyquist plots can be analyzed by using an ideal equivalent electrical circuit consisting of resistance and capacitance. This circuit can set up a connection between the microstructure and physical properties. The Nyquist plots at different temperatures have been well fitted with an equivalent circuit [29,30]. As shown in the inset of Figure 5b, the circuit consists of two sub-circuits in series. (*C_gb_*, *C_g_*) and (*R_gb_*, *R_g_*) represent the capacitances and resistances of grain boundaries and grains, respectively. CPE denotes a constant phase element with an impedance Z^*^_CPE_ = *A(jù)^n^*, where *A* is the scale factor and *n* decides the departure from an ideal capacitor. Figure 5b shows a representative result at 235 K and Table 1 provides the fitted parameters. The circuit made up of two sub-circuits in series indicates that there are two relaxations [30]. The relaxation at low frequency is related to grain boundaries and the one at high frequency is duo to the grains [4,31]. The electrode has little influence on the dielectric properties, which is consistent with the above conclusion. As shown in Table 1, the resistance of the grain is smaller than that of grains boundaries, which is similar to the results of reference [1].

**Figure 6:**
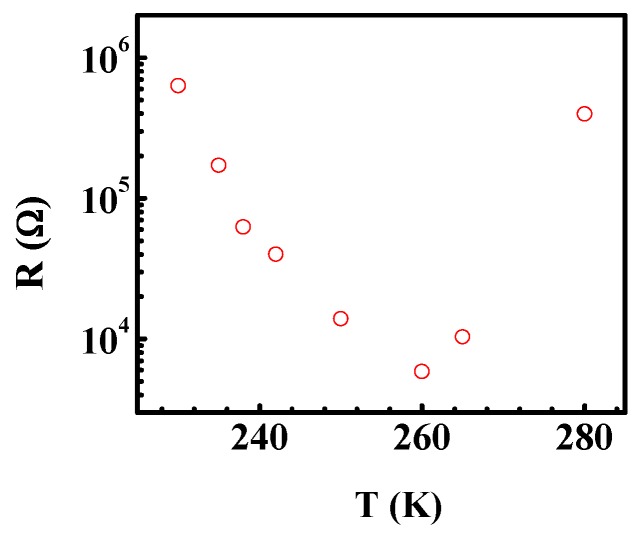
(Color online) The resistance R versus the temperature.

**Figure 7:**
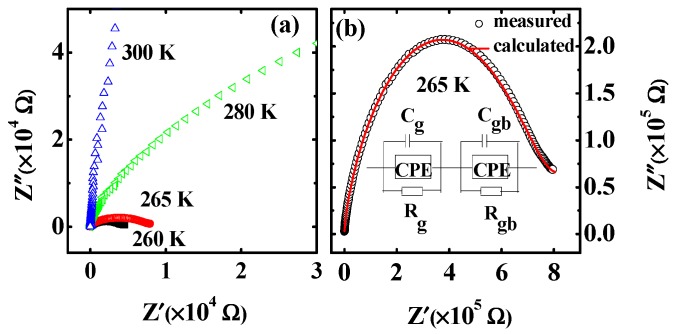
(Color online) (**a**) Complex impedance above 260 K, (**b**) Nyquist plots for the circuit at 265 K.

It is necessary to clarify the origin of the abnormal dielectric relaxation *A_M2_* shown in Figure 3. It is well-known that the Vogel-Fulcher relation, the Arrhenius relation, or a complicated relaxation time distribution function is usually used to derive the relaxation time for the normal thermally activated phenomena [20,32]. It is difficult to understand that the relaxation behavior that the peak position shifts to low temperature with the frequency increasing for the abnormal thermally activated behavior, as shown in Figure 3. Similar phenomena were shown in BaTiO_3_ [3], Gd_2_SiO_5_ laser crystals [20], and BaTi_0.85_Zr_0.15_O_3_ ceramics [33], which are related to the PTCR effect. Therefore, the abnormal dielectric behavior in Mn_3_O_4_ might be associated with the PTCR effect in a similar way to for the above materials. The resistance R of Mn_3_O_4_ polycrystalline at different temperatures was derived according to the impedance spectrum (*Z”*–*f*) studies, since the impedance peak intensity yields the value of *R*/2 [3]. Figure 6 displays the temperature dependence of the resistance (*R*). As expected, there is a critical point at 260 K. The resistance decreases with the increasing of temperature below 260 K, and increases above 260 K. The results show there is a positive temperature coefficient resistor above 260 K (the PTCR effect). The temperature region of the PTCR effect and that of the abnormal dielectric phenomenon matches perfectly. This result implies that the abnormal dielectric phenomenon stems from the PTCR effect in Mn_3_O_4_ polycrystalline. We also studied the Nyquist plots above 260 K. The semicircular arc radius of the Nyquist plot decreases as the temperature decreases (shown in Figure 7a), which shows that the resistance of the Mn_3_O_4_ ceramics increases with the temperature. The impedance data can also be fitted with the equivalent circuit, as shown in Figure 7b, and Table 1 gives the fitted parameters at 265 K. The resistance of the grain boundaries *R_gb_* is about 9 × 10^15^ Ù, which is much larger than that of the grain. According to the Heywang-Jonker model, the PTCR effect can be explained by the difference of the resistances between the grain and grain boundary.

**Figure 8:**
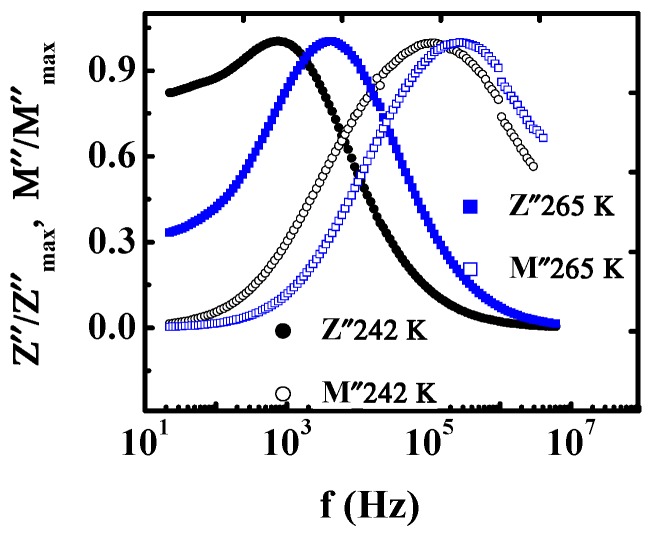
(Color online) Normalized functions of electric modulus and impedance versus frequency at 242 and 265 K.

The normalized functions of *M″/M″_max_* and *Z″/Z″_max_* are shown in Figure 8 measured at 242 and 265 K. For the same temperature, the *Z″/Z″_max_* and *M″/M″_max_* peaks locate near to each other but not overlap. As reference [34] states, the overlapping of the peak position of *M″/M″_max_* and *Z″/Z″_max_* curves or not is a criterion of delocalized or long-range motions of charge carriers. Therefore, there are long-range and localized conduction below and above 260 K for the Mn_3_O_4_ polycrystalline.

## 4. Conclusions

In summary, the temperature and frequency dependences of dielectric constant/electric modulus/impedance spectrums have been investigated in a Mn_3_O_4_ polycrystalline sample. There are two types of dielectric relaxations. The low-temperature relaxation is due to the hopping of oxygen vacancies. The other dielectric relaxation occurs above 260 K and is different from the general thermal activation behavior, where the resistance increases with the increasing of the temperature. The temperature region of the PTCR effect and that of the abnormal dielectric behavior matches perfectly with each other. This result implies that the abnormal dielectric behavior can be ascribed to the PTCR effect in Mn_3_O_4_ polycrystalline. In line with the normalized functions of electric modulus and impedance spectrums, it can be concluded that there are long-range and localized forms of conduction below and above 260 K for Mn_3_O_4_ polycrystalline.

## Figures and Tables

**Table 1:** The fitting parameters obtained according to the experimental data by the equivalent circuit.

Temp.(K)	*R_gb_* (MΩ)	*C_gb_* (pF)	CPE (10^−8^ S·s^n^)	*n*	*R_g_* (MΩ)	*C_g_* (pF)	CPE (10^−8^ S·s^n^)	*n*
235	2.628	189.3	24.54	0.473	0.951	108.2	1.501	0.564
265	9 × 10^9^	1081	2738	0.278	0.074	71.95	3.926	0.568
